# Prediction of gestational age using urinary metabolites in term and preterm pregnancies

**DOI:** 10.1038/s41598-022-11866-6

**Published:** 2022-05-16

**Authors:** Kévin Contrepois, Songjie Chen, Mohammad S. Ghaemi, Ronald J. Wong, Fyezah Jehan, Sunil Sazawal, Abdullah H. Baqui, Jeffrey S. A. Stringer, Anisur Rahman, Muhammad I. Nisar, Usha Dhingra, Rasheda Khanam, Muhammad Ilyas, Arup Dutta, Usma Mehmood, Saikat Deb, Aneeta Hotwani, Said M. Ali, Sayedur Rahman, Ambreen Nizar, Shaali M. Ame, Sajid Muhammad, Aishwarya Chauhan, Waqasuddin Khan, Rubhana Raqib, Sayan Das, Salahuddin Ahmed, Tarik Hasan, Javairia Khalid, Mohammed H. Juma, Nabidul H. Chowdhury, Furqan Kabir, Fahad Aftab, Abdul Quaiyum, Alexander Manu, Sachiyo Yoshida, Rajiv Bahl, Jesmin Pervin, Joan T. Price, Monjur Rahman, Margaret P. Kasaro, James A. Litch, Patrick Musonda, Bellington Vwalika, The Alliance for Maternal and Newborn Health Improvement (AMANHI), Fyezah Jehan, Sunil Sazawal, Abdullah H. Baqui, Muhammad I. Nisar, Usha Dhingra, Rasheda Khanam, Muhammad Ilyas, Arup Dutta, Usma Mehmood, Saikat Deb, Aneeta Hotwani, Said M. Ali, Sayedur Rahman, Ambreen Nizar, Shaali M. Ame, Sajid Muhammad, Aishwarya Chauhan, Waqasuddin Khan, Rubhana Raqib, Sayan Das, Salahuddin Ahmed, Tarik Hasan, Javairia Khalid, Mohammed H. Juma, Nabidul H. Chowdhury, Furqan Kabir, Fahad Aftab, Abdul Quaiyum, Alexander Manu, Sachiyo Yoshida, Rajiv Bahl, The Global Alliance to Prevent Prematurity and Stillbirth (GAPPS), Anisur Rahman, Jesmin Pervin, Joan T. Price, Monjur Rahman, Margaret P. Kasaro, James A. Litch, Patrick Musonda, Bellington Vwalika, Jeffrey S. A. Stringer, Gary Shaw, David K. Stevenson, Nima Aghaeepour, Michael P. Snyder

**Affiliations:** 1 Department of Genetics, Stanford University School of Medicine, Stanford, CA USA; 2 Stanford Cardiovascular Institute, Stanford University, Stanford, CA USA; 3 Department of Anesthesiology, Perioperative and Pain Medicine, Stanford University School of Medicine, Stanford, CA USA; 4 Department of Pediatrics, Division of Neonatal and Developmental Medicine, Stanford University School of Medicine, Stanford, CA USA; 5 Digital Technologies Research Centre, National Research Council Canada, Toronto, ON Canada; 6 Department of Pediatrics and Child Health, Aga Khan University, Karachi, Pakistan; 7 Biorepository and Omics Research Group, Faculty of Health Sciences, Medical College, Aga Khan University, Karachi, Pakistan; 8 Center for Public Health Kinetics, New Delhi, India; 9 Public Health Laboratory IdC, Pemba, Zanzibar, Tanzania; 10 International Center for Maternal and Newborn Health, Department of International Health, Johns Hopkins Bloomberg School of Public Health, Baltimore, MD USA; 11 Projahnmo Research Foundation, Abanti, Banani, Dhaka, Bangladesh; 12 International Center for Diarroheal Disease Research, Mohakhali, Dhaka, Bangladesh; 13 Maternal, Newborn, Child and Adolescent Health Research, World Health Organization, Geneva, Switzerland; 14 Maternal and Child Health Division, International Centre for Diarrhoeal Disease Research, Dhaka, Bangladesh; 15 Department of Obstetrics and Gynecology, University of North Carolina at Chapel Hill, Chapel Hill, NC USA; 16 UNC Global Projects Zambia, Lusaka, Zambia; 17 Global Alliance to Prevent Prematurity and Stillbirth, Seattle, USA; 18 Department of Biostatistics, University of Zambia, Lusaka, Zambia; 19 Department of Obstetrics and Gynecology, University of Zambia School of Medicine, Lusaka, Zambia

**Keywords:** Molecular medicine, Predictive markers

## Abstract

Assessment of gestational age (GA) is key to provide optimal care during pregnancy. However, its accurate determination remains challenging in low- and middle-income countries, where access to obstetric ultrasound is limited. Hence, there is an urgent need to develop clinical approaches that allow accurate and inexpensive estimations of GA. We investigated the ability of urinary metabolites to predict GA at time of collection in a diverse multi-site cohort of healthy and pathological pregnancies (n = 99) using a broad-spectrum liquid chromatography coupled with mass spectrometry (LC–MS) platform. Our approach detected a myriad of steroid hormones and their derivatives including estrogens, progesterones, corticosteroids, and androgens which were associated with pregnancy progression. We developed a restricted model that predicted GA with high accuracy using three metabolites (rho = 0.87, RMSE = 1.58 weeks) that was validated in an independent cohort (n = 20). The predictions were more robust in pregnancies that went to term in comparison to pregnancies that ended prematurely. Overall, we demonstrated the feasibility of implementing urine metabolomics analysis in large-scale multi-site studies and report a predictive model of GA with a potential clinical value.

## Introduction

Human pregnancy involves a myriad of interconnected biological processes that are precisely regulated to ensure proper fetal development and growth^1^. A reliable estimation of gestational age (GA) is critical to provide optimal care for the expectant mother and inform clinical decisions, especially in pregnancies with pathological conditions such as intrauterine growth restriction (IUGR) and preterm birth (PTB)^2^. In current clinical practice, GA is best estimated by fetal ultrasound performed before 13 weeks of gestation^3^. However, early ultrasound is often not feasible in resource-limited settings due to later presentation to care or lack of equipment and trained sonographers^4^. Alternatively, GA can be estimated using the reported first day of the last menstrual period (LMP) or various maternal and fetal biometrics, but these methods have been shown to be imprecise or even biased^5^, stressing the need to develop novel ways to estimate GA. Misclassifications of GA can result in inaccurate estimations of prematurity, a major cause of neonatal mortality in South Asia and sub-Saharan Africa^6^. The study of risk factors of prematurity and its impact on long-term outcomes is also impeded by the absence of reliable measures of GA.

Recent omic studies performed in blood have successfully characterized the timing of biological processes during healthy pregnancy and revealed precisely-tuned chronological changes at the level of maternal cell-free RNA^7^, immune cells^8^, plasma proteins^9^, and metabolites^10,11^. These observations have unveiled a potential utility of blood molecular constituents towards more accurate estimations of GA. While most omic layers demonstrated predictive value, metabolomics—the comprehensive study of metabolites—was among the most performant with steroid hormones and their derivatives being the most predictive^10,11^. Despite the many advantages of urine as a clinical sample (e.g. non-invasive collection, sterile, and largely-free from interfering proteins and complex lipids), the feasibility of predicting GA using urinary metabolite levels remains unexplored.

In this context, we profiled metabolites using an untargeted liquid chromatography coupled with mass spectrometry (LC–MS) platform in urine samples collected in early pregnancy (8–19 weeks) from women across multiple international study sites. Using random forest (RF) machine learning, we demonstrated that a small subset of urinary metabolites can predict GA with high precision and accuracy. Metabolites selected in the model informed on individual molecules and biological processes that associated with pregnancy progression. We found that GA was not predicted as accurately among women who went on to deliver preterm, which was explained in part by a larger inter-individual variability of predictive metabolites in this population.

## Results

### Research design and metabolic coverage

**Figure 1:**
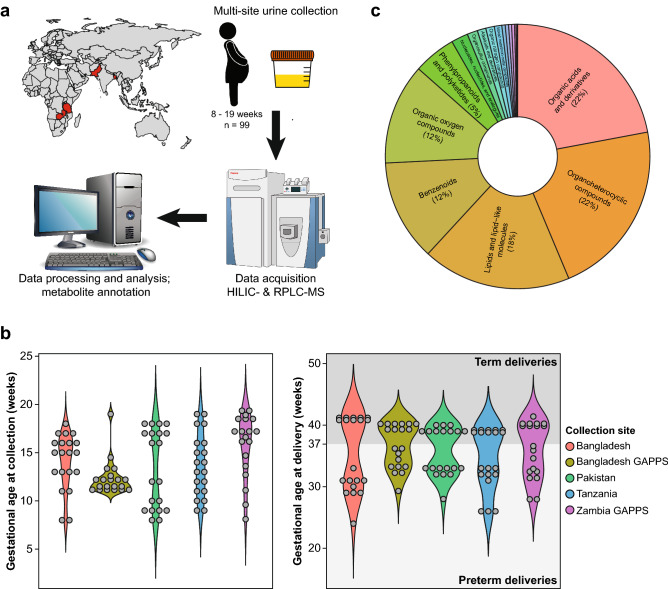
Study design and cohort characteristics. (**a**) Urine samples from 99 pregnant women were collected across 5 sites and analyzed using a broad-spectrum metabolomics LC–MS platform. The sources of the images are described in the Methods section. (**b**) GA at collection and at delivery across the collection sites. Urine samples were collected early in pregnancy 8–19 weeks and 49 women delivered at term (> 37 weeks’ GA) and 50 women delivered preterm (≤ 37 weeks’ GA). (**c**) Structural categorization of detected urine metabolites according to the “Superclass level” of the ClassyFire classification system.

A total of 99 urine samples from term (≥ 37 weeks’ GA, n = 49) and preterm (< 37 weeks’ GA, n = 50) pregnancies collected between 8 and 19 weeks of gestation were selected from each of the five AMANHI and GAPPS sites in Asia and Africa (Fig. 1a,b). Participant demographics and birth characteristics are presented in Table 1. Urinary metabolites were profiled using an untargeted metabolomics platform that combines hydrophilic interaction chromatography (HILIC) and reverse phase liquid chromatography (RPLC) coupled with high resolution mass spectrometry^12^. After data processing and curation, 6630 metabolic features representing a wide chemical diversity were retained, including organic acids (22%), organoheterocyclic compounds (22%), lipids and lipid-like molecules (18%), benzenoids (12%), organic oxygen compounds (12%) and other minor chemical classes (Fig. 1c and Table S1). A large proportion (21%) of lipids and lipid-like molecules were steroid hormones, which is expected for samples collected during pregnancy.

**Table 1:** Demographics and birth characteristics.

	All n = 99	Term n = 49	Preterm n = 50	Validation n = 20
**Demographics**				
Maternal age (years)	24.7 ± 5.2	24.9 ± 4.5	24.6 ± 5.9	31.9 ± 4.8
BMI (kg/m^2^)	22.4 ± 3.9 (n = 90)	22.4 ± 3.7 (n = 44)	22.4 ± 4.1 (n = 46)	22.4 ± 3.0 (n = 20)
Parity	1.5 ± 1.5 (n = 93)	1.6 ± 1.5 (n = 48)	1.4 ± 1.5 (n = 45)	0.7 ± 0.8 (n = 20)
Smoker	0/81 (0.0%)	0/42 (0.0%)	0/39 (0.0%)	0/20 (0.0%)
History of stillbirth	10/90 (11.1%)	7/45 (15.6%)	3/45 (6.7%)	0/20 (0.0%)
History of preterm birth	45/82 (54.9%)	26/44 (59.1%)	19/38 (50.0%)	3/20 (0.0%)
Eclampsia	1/92 (1.1%)	0/49 (0.0%)	1/43 (2.3%)	0/20 (0.0%)
Preeclampsia	4/99 (4.0%)	1/49 (2.0%)	3/50 (6.0%)	0/20 (0.0%)
Gestational hypertension	7/93 (7.5%)	1/49 (2.0%)	6/44 (13.6%)	0/20 (0.0%)
Gestational diabetes	0/84 (0.0%)	0/44 (0.0%)	0/40 (0.0%)	0/20 (0.0%)
**Birth characteristics**				
Gestational age at delivery	35.7 ± 4.6	39.9 ± 0.8	31.7 ± 2.8	39.6 ± 1.2
Gender of child				
Male	49/98 (50.0%)	22/49 (44.9%)	27/49 (55.1%)	12/20 (60.0%)
Female	49/98 (50.0%)	27/49 (55.1%)	22/49 (44.9%)	8/20 (40.0%)

Values are mean ± standard deviation.

### Urine metabolomics data quality and effect of collection site

The quality of the dataset was first examined to ensure technical reproducibility and the absence of a batch effect (Figure S1a). Pooled samples (QC) clustered together and samples analyzed in different batches were intermixed on principal component analysis (PCA) plots. In addition, replicate samples from distinct aliquots processed and analyzed in a random order (n = 172 from 99 samples) clustered together, indicating high reproducibility and robustness of the metabolomic platform (Figure S1b). Urine concentrations can vary substantially depending on the hydration state of the participant. This can be visualized in Figure S1c with a variable distribution of MS signal intensity detected in each individual. We applied probabilistic quotient normalization (PQN) that successfully eliminated the dilution effect.

A main concern when collecting samples from different sites is the variability in metabolite levels induced by differential sample collection (i.e. time of collection, fasting status, clean catch) and handling (i.e. timing of processing, freezing and transportation) procedures. This is especially true for those metabolites that are susceptible to enzymatic activity and degradation. Urine samples collected at different sites were mostly overlapping on a PCA plot, suggesting minor site-specific collection effects (Figure S1d) and validates the standard operating procedure followed by the different sites. Hence, all the samples provided for this analysis could be used together to investigate the ability of urinary metabolites to predict GA.

### Prediction of gestational age at time of collection

**Figure 2:**
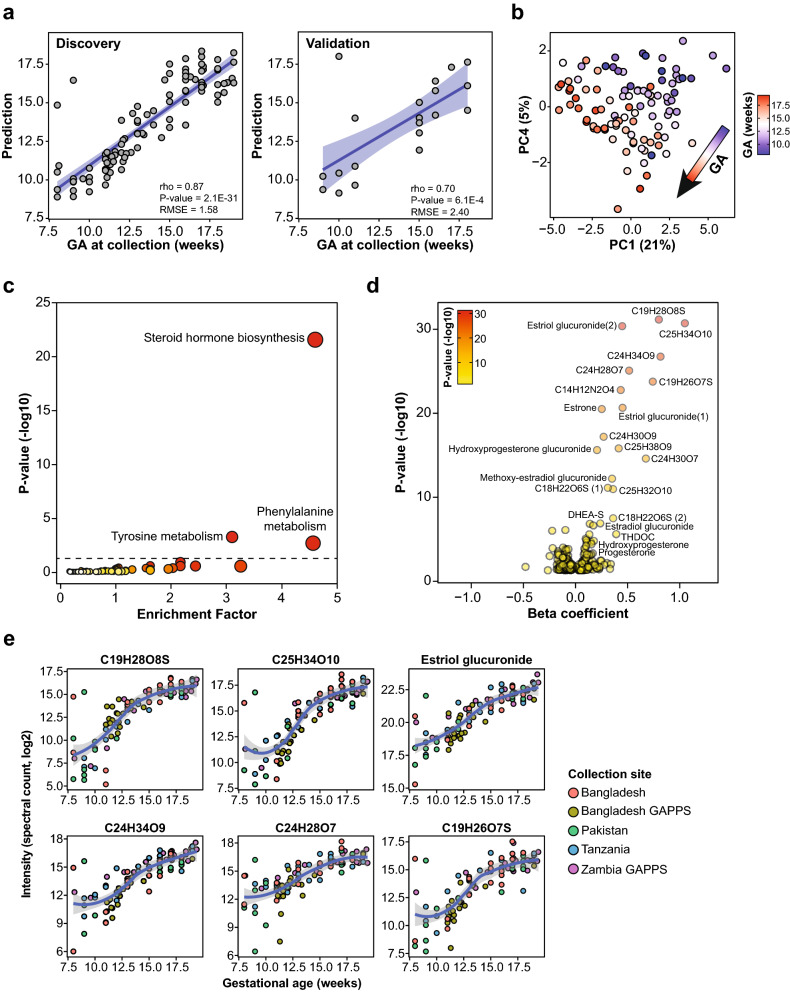
Prediction of gestational age at time of collection and associated biological processes. (**a**) Performance of the restricted RF prediction model of GA that uses three metabolites (C19H26O7S, C24H30O9 and estriol glucuronide) and all the samples in the study (n = 99). The model was validated in an independent cohort (n = 20). The blue area represents the 95% confidence interval. (**b**) Principal component analysis using predictive metabolites (*P*-value < 0.05). PC1 and PC4 were chosen because they associated the most strongly with GA. (**c**) KEGG metabolic pathway enrichment analysis. (**d**) Volcano plot of annotated significant metabolites (*P*-value < 0.05). Beta coefficients were calculated using a linear modeling and *P*-values were calculated from Spearman correlations. (**e**) Top 6 metabolites in the predictive model and LOESS fit across all the samples. The grey area represents the 95% confidence interval.

We next investigated whether urine metabolites could be used to accurately predict GA at time of collection. A random forest (RF) algorithm was employed using all 6630 metabolic features and yielded a model that could predict GA with a cross-validated Spearman coefficient of correlation of 0.83 (*P*-value = 2.4E−26 and a root mean squared error (RMSE) = 1.79 weeks) (Figure S2a and Table S2). However, urine metabolite levels were not successful in predicting GA at delivery (Figure S2b). For potential use in a field setting, we generated a restricted model using the least number of metabolites while retaining predictive ability. The optimal model included three metabolites and yielded excellent predictive ability (rho = 0.87, *P*-value = 2.1E−31 and RMSE = 1.58 weeks) (Fig. 2a). This parsimonious model was validated using samples from an independent cohort of uncomplicated pregnancies (n = 20, rho = 0.70, *P*-value = 6.1E−04 and RMSE = 2.40 weeks). The validation cohort was enrolled at the Lucile Packard Children’s Hospital at Stanford University and was composed of white non-Hispanic women suggesting that the model is valid in diverse ethnicities and environments (Table 1). Among three metabolites selected in the model two were uncharacterized molecules with steroid-like structures (C19H28O8S and C25H34O10) and one was an estrogen (estriol glucuronide). It should be noted that GA for two samples were overestimated by the model. This was explained by an overcorrection of the MS signal by the normalization procedure for these samples that were the most diluted in the study.

Biological processes tracking with pregnancy progression were then investigated by using metabolites significantly associated with GA (Spearman's rank order correlation, 752/6630 with *P*-value < 0.05). The timing of sampling could be visualized using the significant metabolites along two dimensions by plotting the principal components (PCs) PC1 and PC4 that were most strongly associated with GA (Fig. 2b). Pathway enrichment analysis revealed that steroid hormone biosynthesis (*P*-value = 2.9E−22) was significantly associated with GA (Fig. 2c) and involved a myriad of steroid hormones and their derivatives, such as estrogen derivatives (e.g. estriol glucuronide, estrone and estradiol glucuronide), progesterone derivatives (e.g. hydroxyprogesterone glucuronide, hydroxyprogesterone and progesterone), corticosteroids (e.g. tetrahydrodeoxycorticosterone [THDOC]) and androgens (e.g. dehydroepiandrosterone sulfate [DHEA-S]) (Fig. 2d and Table S3). As expected, all of these molecules were positively associated with GA (Fig. 2e). In addition, many uncharacterized molecules with steroid-like structures were strongly associated with GA including sulfated molecules (e.g. C19H28O8S and C19H26O7S) and potential glucuronide derivatives (e.g. C25H34O10 and C24H34O9). Even though most significant metabolites were positively associated with GA (55%), a large proportion presented a negative association (45%) (Fig. 2d and Figure S2c). In addition to the steroid pathway, tyrosine (*P*-value = 5.9E−04) and phenylalanine metabolism (*P*-value = 2.2E−03) were moderately associated with GA. Metabolites belonging to significant pathways were visualized on a KEGG map (Figure S2d).

### Differential gestational age prediction in term and preterm cohorts

**Figure 3:**
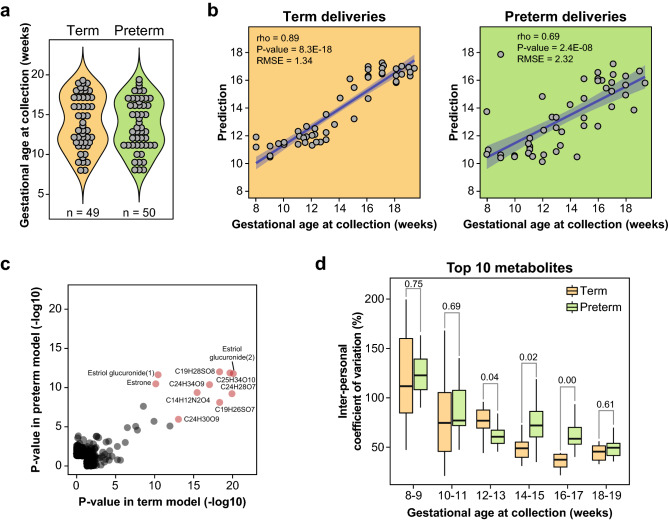
Prediction of gestational age at time of collection in term and preterm pregnancies. (**a**) Distribution of GA at sample collection in term (n = 49) and preterm pregnancies (n = 50). (**b**) Performance of the RF prediction models of GA in term and preterm deliveries. (**c**) *P*-values of selected metabolites in term and preterm RF models. Metabolites that are most predictive tend to be significant in both models. The top 10 metabolites are represented in red. (**d**) Coefficient of variation of the top 10 metabolites across GA ranges.

Next, we sought to investigate GA prediction in samples collected from women who would go on to deliver at term (n = 49) versus preterm (n = 50, < 37 weeks’ GA). GA at collection did not differ between the two groups (term: 13.60 weeks [11.50–17.00], preterm: 13.35 weeks [11.10–16.52], *P*-value = 0.64) (Fig. 3a). The RF algorithm yielded a model that performed better among the term deliveries (rho = 0.89, *P*-value = 8.3E−18 and RMSE = 1.34 weeks) than among the preterm deliveries (rho = 0.69, *P*-value = 2.4E−08 and RMSE = 2.32 weeks) (Fig. 3b). Most metabolites selected in these models were also significant in the model that used all samples, with 66% and 60% overlap in term and preterm models, respectively (Table S2). The metabolites driving both term and preterm models were identical however, they were more strongly correlated with GA in the term cohort as indicated by their smaller *P*-values (Fig. 3c). This was not explained by differential metabolite trajectories or abundances but rather by a higher inter-individual variability of their absolute levels between weeks 14 and 17 (Fig. 3d and Figure S3a–c). Even though the top metabolites selected in both models were the same, the most important metabolites differed with estrogens (estrone and estriol glucuronide) and uncharacterized metabolites (C19H26O7S and C24H30O9) being more important in the preterm and the term models, respectively (Figure S3d).

**Figure 4:**
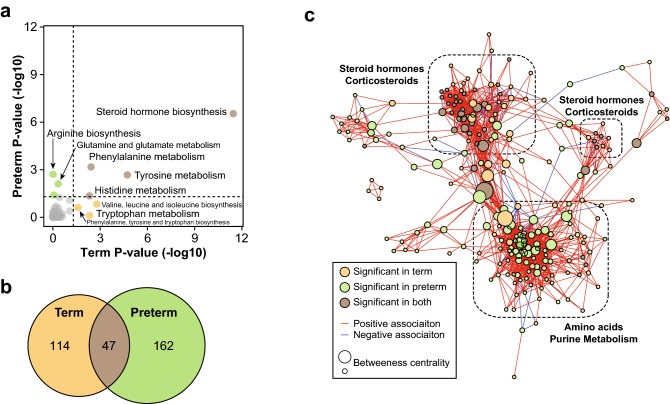
Biological processes associated with term and preterm prediction models. (**a**) KEGG metabolic pathway enrichment analysis using metabolites selected in the term and preterm RF models (*P*-value < 0.05). (**b**) Venn diagram of validated metabolites predictive of GA (*P*-value < 0.05) in term and preterm models. (**c**) Pairwise spearman correlation network. Nodes were color-coded by model significance and their size represents the betweenness centrality.

Pathway enrichment analysis confirmed the results from the general model with significant enrichment of steroid hormone biosynthesis, and phenylalanine and tyrosine metabolism in both models (Fig. 4a). Interestingly, certain pathways were enriched exclusively in the term and preterm models. Valine, leucine and isoleucine biosynthesis (*P*-value = 1.6E−03) as well as tryptophan metabolism (*P*-value = 3.7E−03) were associated with GA in term pregnancies, while arginine biosynthesis (*P*-value = 1.9E−03) and glutamine and glutamate metabolism (*P*-value = 7.7E−03) were associated with GA in preterm pregnancies. Correlation network analysis revealed two clusters of highly correlated metabolites (Fig. 4b,c). One cluster was composed of steroid hormones, with a majority of metabolites selected in both models. A second cluster was mostly composed of amino acids (9/20 amino acids including 3 branched chain amino acids as well as acetylated amino acids) and purine metabolites (purine nucleosides guanosine and inosine as well as their methylated forms), and was exclusively selected in the preterm model. These differences may reflect dysregulated biological processes associated with PTB.

## Discussion

In this work, we show that urinary metabolites can accurately predict GA at time of collection from samples collected in the first and early second trimesters of healthy and pathological pregnancies from diverse geographies. Our predictions were more robust in pregnancies that went to term. These findings are in line with recent reports showing that maternal blood metabolites can successfully predict GA^10,11^. Our method provides a simpler alternate for GA dating using urine, which can be collected non-invasively and requires minimal processing^13^. This is in contrast to blood that requires specific collection, handling and processing to retain sample integrity.

We also show that implementing standard operating procedures for urine collection across sites is feasible without site effects by utilizing global metabolic profiling^12^. Our LC–MS approach was robust and sensitive with the detection of a wide variety of chemicals belonging to 187 “Superclass level” of the ClassyFire classification system.

Regression RF selected a set of urine metabolites that accurately predicted GA. Steroid hormones and their derivatives including estrogens, progesterones, corticosteroids and androgens were among the strongest predictors. For instance, we detected progesterone and hydroxyprogesterone that have previously been shown to be strongly associated with the length of gestation and are widely recommended for women at high risk for PTB in countries with a very high human development index^14^. The level of THDOC, estriol glucuronide, progesterone, and DHEA-S were among the top predictors in urine mirroring recent findings in plasma^11^. The roles of progesterone, estriol glucuronide, and DHEA-S in pregnancy are well described^15^, however, neurosteroid THDOC has been less studied^16^. These molecules present value to monitor the length of pregnancy and may also prove useful to detect pregnancy conditions such as prenatal stress^17^ and their impact on pregnancy outcome and long-term infant health and development^18^.

Our untargeted metabolomics platform also detected many uncharacterized molecules that were defined by their elemental composition. Interestingly, many of these molecules were associated with GA at sampling and hold a higher predictive ability than many molecules previously described in the literature. For example, 7 of the top 10 metabolites were uncharacterized with C19H28O8S and C25H34O10 being the two most predictive analytes. These molecules are likely conjugated steroids with the former containing a sulfate and the latter a glucuronic acid moiety. Conjugated molecules are abundant in urine since conjugation increases their solubility and facilitates urinary excretion^19^. These results highlight the value of untargeted LC–MS metabolomics approaches for the sensitive and simultaneous profiling of many steroid metabolites and derivatives giving insights into steroid biosynthesis and excretion processes.

We also present a restricted model that uses the abundance of only three metabolites and show that GA can be estimated early in pregnancy with better accuracy (RMSE = 1.6 weeks) than models developed in blood using cell-free RNA (RMSE = 4.3 weeks)^20^ or metabolites (RMSE = 2.5 weeks)^11^. Importantly, the restricted model was generalizable when applied to an independent cohort of uncomplicated pregnancies. In contrast to recent studies that identified molecular signatures associated with GA using multiple samples per pregnancy collected at a single site^10,11^, we demonstrate that urinary metabolites can accurately estimate GA as compared to ultrasound dating using a single time point per pregnancy from populations across multiple countries. Additional studies on larger cohorts will be necessary for final demonstration of feasibility. In this study, urine samples were frozen upon collection and kept at − 80 °C until analysis, hence, follow up studies will be necessary to assess the stability of the three predictive metabolites to allow storage and/or transportation at 4 °C or at ambient temperature. With the objective of developing a clinical test, the two unknown metabolites C19H28O8S and C25H34O10 will need to be fully characterized before being able to develop a simple and cost-effective dipstick test. Finally, it remains to be determined if the model performs well on samples collected before week 8 and after week 19. Prior research on plasma cell free RNA indicated that prediction accuracy increased with gestation^20^ so it may hold true for our model as well. This remains a critical area of research, since many women in the settings where this technology would be most useful do not present for pregnancy care until 20 weeks of gestation or more^21^. Of note, we were not successful in generating a model that could distinguish pregnancies destined to deliver at term from those destined to deliver prematurely presumably because of the relatively small sample size. The investigation of early markers of prematurity using larger cohorts is under way.

Regression RF prediction models were also generated to predict GA in samples from mothers that delivered term and preterm (< 37 weeks’ GA). Even though the same metabolites (i.e. steroid hormones) were the most predictive in both models, the prediction performance was higher for term deliveries. This observation may in part reflect a tighter control of the level of these molecules in term pregnancies rather than a difference in their absolute abundance. Correlation network analysis revealed a cluster of amino acids and purine metabolites mainly selected in the preterm model encompassing differences in these pathways in term and preterm pregnancies. Many of these molecules have been reported as being dysregulated in PTB including choline, dimethylarginine, methionine, phenylalanine, tryptophan, valine, threonine, isoleucine, leucine and xanthine^22^. Targeted and untargeted metabolomics approaches have been employed to study PTB and have identified various early biomarkers. However, very little consensus has yet emerged owing to varying maternal sample sources (i.e. cervicovaginal fluid, amniotic fluid, blood and urine), GA at sampling and participant demographics. Our study is limited by the fact that 59% of the patients with a term pregnancy had a history of a preterm birth. This is an obstetrical high-risk group which cannot be generalized to a larger population.

In conclusion, our study demonstrated that a small set of urinary metabolites can predict GA using a single sample in a diverse cohort. Our work paves the way for a simple dipstick test that could be implemented in under-resourced or remote settings where sonography is not feasible or affordable.

## Methods

### Study sites and IRB consent

The study involves five cohorts from Asia and Africa as part of an international consortium, ‘Multi-omics for Mothers and Infants’ (MOMI). The three cohort sites, from the Alliance for Maternal and Neonatal Health Improvement (AMANHI) biorepository study are located in Sylhet (Bangladesh), Karachi (Pakistan), and Pemba (Tanzania). The two cohorts from the Global Alliance to Prevent Prematurity and Stillbirth (GAPPS) consortium are located in Matlab (Bangladesh, Preterm and Stillbirth Study [PreSSMat]) and Lusaka (Zambia, Zambian Preterm Birth Prevention Study [ZAPPS]). The primary objective of the AMANHI study is to establish a biorepository towards discovery of biomarkers of adverse pregnancy-related outcomes^23^. PreSSMat is a prospective cohort study designed to assess biological, environmental, and social determinants of adverse pregnancy outcomes^24^ while ZAPPS is a prospective cohort study and biorepository designed to characterize the factors associated with preterm birth and outcomes in Zambia^25^.

### Ethics declarations

The AMANHI study received ethical approval from the World Health Organization (WHO) Ethics Review Committee as well as local and institutional ethics committees for all three sites: icddr,b and John Hopkins University for Bangladesh, Aga Khan University for Pakistan and Zanzibar Medical Research and Ethics Committee (ZAMREC) and John Hopkins University for Tanzania. The ZAPPS cohort was approved by relevant authorities at both the University of Zambia School of Medicine and the University of North Carolina at Chapel Hill. PreSSMat received approval from the Research and Ethical Review Committees of the International Centre for Diarrhoeal Disease Research in Bangladesh (PR-14067). Informed consent for participation in the original study and for future research use of specimens was obtained from each woman prior to enrollment. The study was also approved by the Stanford Institutional Review Board (IRB 21956). All experiments were performed in accordance with relevant guidelines and regulations.

### Study design

Ninety-nine pregnant women were selected for the study and included 20 participants from each site with half delivering preterm (< 37 weeks’ GA) and half delivering at term (≥ 37 weeks’ GA). Only 9 samples were provided from term pregnancies at the Zambia site. Women with multiple births, congenital malformations, stillbirth, or induction of labor for any cause were excluded. Outcomes were assessed through either study procedures on the labor ward or, among those delivering elsewhere, through participant interview via direct phone calls, household visits, and/or medical record review at a postnatal visit.

### Urine collection and gestational age assessment

The study was comprised of a single urine sample for each participant (n = 99) that was collected at a prenatal visit after ultrasound confirmed at < 20 weeks of gestation. Ultrasound imaging was performed by trained sonologists and GA was estimated following guidelines from the American College of Obstetricians and Gynecologists^3^ (Bangladesh GAPPS ) and using INTERGROWTH-21st equations^26^ (Zambia) or Hadlock's formulas^23,27^ (AMANHI sites: Bangladesh, Pakistan, Tanzania). GA was reported in weeks. All study sites employed a uniform method for urine collection and handling. Urine samples were collected at any time of the day, aliquoted and frozen at − 80 °C within 2 h of collection. Deidentified urine aliquots were shipped on dry ice from each biorepository to Stanford University as a single batch and under continuous temperature monitoring. Urine samples from 20 uncomplicated pregnancies collected between 8 and 19 weeks of gestation at the Lucile Packard Children’s Hospital at Stanford University, served as the validation cohort.

### Untargeted metabolomics of urine by liquid chromatography (LC)–mass spectrometry (MS)

LC–MS-grade solvents and mobile phase modifiers were obtained from Fisher Scientific (water, acetonitrile, methanol) and Sigma − Aldrich (acetic acid, ammonium acetate). Urine samples were analyzed using a broad-spectrum metabolomics platform consisting of hydrophilic interaction chromatography (HILIC) and reverse phase liquid chromatography (RPLC)–MS^12^.

#### Sample preparation

Frozen urine samples were thawed on ice and centrifuged at 17,000*g* for 10 min at 4 °C. Supernatants (25 µl) were then diluted 1:4 with 75% acetonitrile and 100% water for HILIC- and RPLC-MS experiments, respectively. Each sample was spiked-in with 15 analytical-grade internal standards (IS). Samples for HILIC-MS experiments were further centrifuged at 21,000*g* for 10 min at 4 °C to precipitate proteins.

#### Data acquisition

Metabolic extracts were analyzed using HILIC and RPLC separations in both positive and negative ionization modes as previously described^12^. Data were acquired on a Thermo Q Exactive HF mass spectrometer equipped with a Heated Electrospray Ionization probe (HESI-II) and operating in full MS scan mode. MS/MS data were acquired at different fragmentation energies (NCE 25, 35 and 50) on pooled samples (QC) consisting of an equimolar mixture of all the samples in the study. HILIC experiments were performed using a ZIC-HILIC column 2.1 × 100 mm, 3.5 μm, 200 Å (Merck Millipore) and mobile phase solvents consisting of 10 mM ammonium acetate in 50/50 acetonitrile/water (A) and 10 mM ammonium acetate in 95/5 acetonitrile/water (B). RPLC experiments were performed using a Hypersil GOLD column 2.1 × 150 mm, 1.9 µm, 175 Å (Thermo Scientific) and mobile phase solvents consisting of 0.06% acetic acid in water (A) and 0.06% acetic acid in methanol (B).

Data quality was ensured by: (1) sample randomization for metabolite extraction and data acquisition, (2) multiple injections of a pooled sample to equilibrate the LC–MS system prior to running the sequence (12 and 6 injections for HILIC and RPLC methods, respectively), (3) spike-in labeled IS during sample preparation to control for extraction efficiency and evaluate LC–MS performance, (4) checking mass accuracy, retention time and peak shape of the IS in each sample and (5) injection of a pooled sample every 10 injections to control for signal deviation over time.

#### Data processing

Data from each mode were independently processed using Progenesis QI software (v2.3) (Nonlinear Dynamics) as recently described^28^. Metabolic features from blanks and that did not show sufficient linearity upon dilution in QC samples (r < 0.6) were discarded. Only metabolic features present in > 2/3 of the samples were kept for further analysis. Inter- and intra-batch variations were corrected by applying locally estimated scatterplot smoothing local regression (LOESS) on pooled samples injected repetitively along the batches (span = 0.75). Data were acquired in four batches for HILIC and RPLC modes. Dilution effects were corrected using probabilistic quotient normalization (PQN)^29^. Missing values were imputed by drawing from a random distribution of low values in the corresponding sample. Multiple aliquots (1 to 4) were analyzed for each sample (n = 172 from 99 unique samples). Data from replicates were aggregated by taking the mean (n = 2) or median (n = 3 to 4). Data from each mode were then merged, producing a dataset containing 6630 metabolic features. Metabolite abundances were reported as spectral counts.

#### Metabolic feature annotation

Peak annotation was first performed by matching experimental m/z, retention time and MS/MS spectra to an in-house library of analytical-grade standards^11^. Remaining peaks were identified by matching experimental m/z and fragmentation spectra to publicly available databases including HMDB (http://www.hmdb.ca/), MoNA (http://mona.fiehnlab.ucdavis.edu/) and MassBank (http://www.massbank.jp/) using the R package ‘metID’ (v0.2.0)^30^. Briefly, metabolic feature tables from Progenesis QI were matched to fragmentation spectra with a m/z and a retention time window of ± 15 ppm and ± 30 s (HILIC) and ± 20 s (RPLC), respectively. When multiple MS/MS spectra match a single metabolic feature, all matched MS/MS spectra were used for the identification. Next, MS1 and MS2 pairs were searched against public databases and a similarity score was calculated using the forward dot–product algorithm which considers both fragments and intensities^31^. Metabolites were reported if the similarity score was above 0.4. Spectra from metabolic features of interest important in random forest models (see below) were further investigated manually to confirm identification and were reported in Table S3. We used the Metabolomics Standards Initiative (MSI) level of confidence to grade metabolite annotation confidence (level 1–level 4). Level 1 represents formal identifications where the biological signal matches accurate mass, retention time and fragmentation spectra of an authentic standard run on the same platform. For level 2 identification, the biological signal matches accurate mass and fragmentation spectra available in one of the public databases listed above. Level 3 represents putative identifications that are the most likely name based on previous knowledge of urine composition. Level 4 consists in unknown metabolites. Some metabolites eluted in multiple peaks and are listed with a number in parenthesis following the metabolite name indicating the order of elution.

### Statistical analysis and visualization

#### Random forest prediction modeling

A random forest algorithm was used to build multivariate prediction models to estimate GA at the time of sample collection using all samples (n = 99), samples from term (n = 49) and samples from preterm deliveries (n = 50). The parameters of the models were optimized using internal cross-validation and an external leave-one-out cross-validation strategy was implemented to test the predictions on the excluded sample. The final results were reported as an aggregate of all blinded predictions. A restricted model containing 3 metabolites was developed and validated using an independent cohort (n = 20, Stanford cohort). Importance of metabolic features were derived from the models while *P*-values were calculated from Spearman correlations.

#### Structural characterization of detected urinary metabolites

Superclass level classification was performed using International Chemical Identifiers (InChI) keys for unique metabolic features (n = 2192) using the ClassyFire Batch search https://cfb.fiehnlab.ucdavis.edu/^32^ (Table S1).

#### Pathway enrichment analysis

We used the Mummichog 1 algorithm^33^ in the web tool MetaboAnalyst 4^34^ to search for enriched pathways. Mummichog leverages the organization of metabolic networks to predict functional activity directly from metabolic feature tables, bypassing metabolite identification. Significance of pathways was determined by the one-sided Fisher exact t-test using KEGG pathways^35–37^. *P*-values ≤ 0.05 were considered significant. Visualization of metabolites belonging to significant pathways on the KEGG map was generated using network explorer tool in MetaboAnalyst 4.

#### Correlation network analysis

Pairwise Spearman’s rank correlations were calculated using the R package ‘Hmisc’ (v3.15–0) and weighted, undirected networks were plotted with ‘igraph’ (v0.7.1). Correlations with Bonferroni adjusted *P*-values ≤ 0.01 were included and displayed via the Fruchterman-Reingold method. Nodes were color-coded by significance in the term and preterm models with node size representing the betweenness centrality.

#### Images

The images in Fig. 1a were obtained as follows: the world map was downloaded from https://www.creativeswall.com/25-free-vector-world-maps/ and edited using adobe illustrator CS6 (v16.0.0), the drawing of the mass spectrometer was obtained from Thermo Scientific, the drawing of a computer was downloaded from https://www.netclipart.com/isee/oTmR_desktop-computer-png-clipart-computer-logo-free-download/ and the silhouette of a pregnant woman was downloaded from http://clipart-library.com/free/pregnant-woman-silhouette-clipart.html.

## Supplementary Information

Supplementary Information 1.

Supplementary Information 2.

Supplementary Information 3.

Supplementary Information 4.

## Data Availability

Raw and processed metabolomics data are hosted on the Metabolomics Workbench under the study ID ST001491.

## References

[1] Soma-Pillay P, Nelson-Piercy C, Tolppanen H, Mebazaa A (2016). Physiological changes in pregnancy. Cardiovasc. J. Afr..

[2] Karl S (2015). Preterm or not—An evaluation of estimates of gestational age in a cohort of women from Rural Papua New Guinea. PLoS ONE.

[3] Committee on Obstetric Practice, the American Institute of Ultrasound in Medicine, and the Society for Maternal-Fetal Medicine (2017). Committee Opinion No 700: Methods for estimating the due date. Obstet. Gynecol..

[4] Kim ET, Singh K, Moran A, Armbruster D, Kozuki N (2018). Obstetric ultrasound use in low and middle income countries: A narrative review. Reprod. Health.

[5] Blencowe H (2013). Born too soon: The global epidemiology of 15 million preterm births. Reprod. Health.

[6] The Alliance for Maternal and Newborn Health Improvement (AMANHI) mortality study group (2018). Population-based rates, timing, and causes of maternal deaths, stillbirths, and neonatal deaths in south Asia and sub-Saharan Africa: A multi-country prospective cohort study. Lancet Glob. Health.

[7] Pan W (2017). Simultaneously monitoring immune response and microbial infections during pregnancy through plasma cfRNA sequencing. Clin. Chem..

[8] Aghaeepour N (2017). An immune clock of human pregnancy. Sci. Immunol..

[9] Aghaeepour N (2018). A proteomic clock of human pregnancy. Am. J. Obstet. Gynecol..

[10] Ghaemi MS (2019). Multiomics modeling of the immunome, transcriptome, microbiome, proteome and metabolome adaptations during human pregnancy. Bioinformatics.

[11] Liang L (2020). Metabolic dynamics and prediction of gestational age and time to delivery in pregnant women. Cell.

[12] Contrepois K, Jiang L, Snyder M (2015). Optimized analytical procedures for the untargeted metabolomic profiling of human urine and plasma by combining hydrophilic interaction (HILIC) and reverse-phase liquid chromatography (RPLC)-mass spectrometry. Mol. Cell Proteom..

[13] Stevens VL, Hoover E, Wang Y, Zanetti KA (2019). Pre-analytical factors that affect metabolite stability in human urine, plasma, and serum: A review. Metabolites.

[14] Chang HH (2013). Preventing preterm births: Analysis of trends and potential reductions with interventions in 39 countries with very high human development index. Lancet.

[15] Kuijper EA, Ket JC, Caanen MR, Lambalk CB (2013). Reproductive hormone concentrations in pregnancy and neonates: A systematic review. Reprod. Biomed. Online.

[16] Reddy DS (2003). Is there a physiological role for the neurosteroid THDOC in stress-sensitive conditions?. Trends Pharmacol. Sci..

[17] Brunton PJ (2016). Neuroactive steroids and stress axis regulation: Pregnancy and beyond. J. Steroid Biochem. Mol. Biol..

[18] Coussons-Read ME (2013). Effects of prenatal stress on pregnancy and human development: Mechanisms and pathways. Obstet. Med..

[19] Schiffer L (2019). Human steroid biosynthesis, metabolism and excretion are differentially reflected by serum and urine steroid metabolomes: A comprehensive review. J. Steroid Biochem. Mol. Biol..

[20] Ngo TTM (2018). Noninvasive blood tests for fetal development predict gestational age and preterm delivery. Science.

[21] Vwalika B (2017). Maternal and newborn outcomes at a tertiary care hospital in Lusaka, Zambia, 2008–2012. Int. J. Gynaecol. Obstet..

[22] Carter RA, Pan K, Harville EW, McRitchie S, Sumner S (2019). Metabolomics to reveal biomarkers and pathways of preterm birth: A systematic review and epidemiologic perspective. Metabolomics.

[23] AMANHI (Alliance for Maternal and Newborn Health Improvement) Bio–banking Study group) (2017). Understanding biological mechanisms underlying adverse birth outcomes in developing countries: Protocol for a prospective cohort (AMANHI bio-banking) study. J. Glob. Health.

[24] Murphy MSQ (2019). Incidental screen positive findings in a prospective cohort study in Matlab, Bangladesh: Insights into expanded newborn screening for low-resource settings. Orphanet. J. Rare Dis..

[25] Castillo MC (2018). The Zambian Preterm Birth Prevention Study (ZAPPS): Cohort characteristics at enrollment. Gates Open Re.s.

[26] Papageorghiou AT (2014). International standards for early fetal size and pregnancy dating based on ultrasound measurement of crown-rump length in the first trimester of pregnancy. Ultrasound Obstet. Gynecol..

[27] Hadlock FP, Shah YP, Kanon DJ, Lindsey JV (1992). Fetal crown-rump length: Reevaluation of relation to menstrual age (5–18 weeks) with high-resolution real-time US. Radiology.

[28] Contrepois K (2020). Molecular choreography of acute exercise. Cell.

[29] Rosen Vollmar AK (2019). Normalizing untargeted periconceptional urinary metabolomics data: A comparison of approaches. Metabolites.

[30] Shen X (2019). Metabolic reaction network-based recursive metabolite annotation for untargeted metabolomics. Nat. Commun..

[31] Stein SE, Scott DR (1994). Optimization and testing of mass spectral library search algorithms for compound identification. J. Am. Soc. Mass. Spectrom..

[32] Blazenovic I (2019). Structure annotation of all mass spectra in untargeted metabolomics. Anal. Chem..

[33] Li S (2013). Predicting network activity from high throughput metabolomics. PLoS Comput. Biol..

[34] Chong J (2018). MetaboAnalyst 4.0: Towards more transparent and integrative metabolomics analysis. Nucleic Acids Res..

[35] Kanehisa M, Goto S (2000). KEGG: Kyoto encyclopedia of genes and genomes. Nucleic Acids Res..

[36] Kanehisa M (2019). Toward understanding the origin and evolution of cellular organisms. Protein Sci..

[37] Kanehisa M, Furumichi M, Sato Y, Ishiguro-Watanabe M, Tanabe M (2021). KEGG: Integrating viruses and cellular organisms. Nucleic Acids Res..

