# Author Correction: Prediction of gestational age using urinary metabolites in term and preterm pregnancies

**DOI:** 10.1038/s41598-022-23715-7

**Published:** 2022-11-17

**Authors:** Kévin Contrepois, Songjie Chen, Mohammad S. Ghaemi, Ronald J. Wong, Fyezah Jehan, Sunil Sazawal, Abdullah H. Baqui, Jeffrey S. A. Stringer, Anisur Rahman, Muhammad I. Nisar, Usha Dhingra, Rasheda Khanam, Muhammad Ilyas, Arup Dutta, Usma Mehmood, Saikat Deb, Aneeta Hotwani, Said M. Ali, Sayedur Rahman, Ambreen Nizar, Shaali M. Ame, Sajid Muhammad, Aishwarya Chauhan, Waqasuddin Khan, Rubhana Raqib, Sayan Das, Salahuddin Ahmed, Tarik Hasan, Javairia Khalid, Mohammed H. Juma, Nabidul H. Chowdhury, Furqan Kabir, Fahad Aftab, Abdul Quaiyum, Alexander Manu, Sachiyo Yoshida, Rajiv Bahl, Jesmin Pervin, Joan T. Price, Monjur Rahman, Margaret P. Kasaro, James A. Litch, Patrick Musonda, Bellington Vwalika, Gary Shaw, David K. Stevenson, Nima Aghaeepour, Michael P. Snyder

**Affiliations:** 1 Department of Genetics, Stanford University School of Medicine, Stanford, CA USA; 2 Stanford Cardiovascular Institute, Stanford University, Stanford, CA USA; 3 Department of Anesthesiology, Perioperative and Pain Medicine, Stanford University School of Medicine, Stanford, CA USA; 4 Department of Pediatrics, Division of Neonatal and Developmental Medicine, Stanford University School of Medicine, Stanford, CA USA; 5 Digital Technologies Research Centre, National Research Council Canada, Toronto, ON Canada; 6 Department of Pediatrics and Child Health, Aga Khan University, Karachi, Pakistan; 7 Biorepository and Omics Research Group, Faculty of Health Sciences, Medical College, Aga Khan University, Karachi, Pakistan; 8 Center for Public Health Kinetics, New Delhi, India; 9 Public Health Laboratory IdC, Pemba, Zanzibar, Tanzania; 10 International Center for Maternal and Newborn Health, Department of International Health, Johns Hopkins Bloomberg School of Public Health, Baltimore, MD USA; 11 Projahnmo Research Foundation, Abanti, Banani, Dhaka, Bangladesh; 12 International Center for Diarroheal Disease Research, Mohakhali, Dhaka, Bangladesh; 13 Maternal, Newborn, Child and Adolescent Health Research, World Health Organization, Geneva, Switzerland; 14 Maternal and Child Health Division, International Centre for Diarrhoeal Disease Research, Dhaka, Bangladesh; 15 Department of Obstetrics and Gynecology, University of North Carolina at Chapel Hill, Chapel Hill, NC USA; 16 UNC Global Projects Zambia, Lusaka, Zambia; 17 Global Alliance to Prevent Prematurity and Stillbirth, Seattle, USA; 18 Department of Biostatistics, University of Zambia, Lusaka, Zambia; 19 Department of Obstetrics and Gynecology, University of Zambia School of Medicine, Lusaka, Zambia

Correction to: *Scientific Reports* https://doi.org/10.1038/s41598-022-11866-6, published online 16 May 2022

In the original version of this Article, Fyezah Jehan, Sunil Sazawal, Abdullah H. Baqui, Jeffrey S. A. Stringer, Anisur Rahman, Muhammad I. Nisar, Usha Dhingra, Rasheda Khanam, Muhammad Ilyas, Arup Dutta, Usma Mehmood, Saikat Deb, Aneeta Hotwani, Said M. Ali, Sayedur Rahman, Ambreen Nizar, Shaali M. Ame, Sajid Muhammad, Aishwarya Chauhan, Waqasuddin Khan, Rubhana Raqib, Sayan Das, Salahuddin Ahmed, Tarik Hasan, Javairia Khalid, Mohammed H. Juma, Nabidul H. Chowdhury, Furqan Kabir, Fahad Aftab, Abdul Quaiyum, Alexander Manu, Sachiyo Yoshida, Rajiv Bahl, Jesmin Pervin, Joan T. Price, Monjur Rahman, Margaret P. Kasaro, James A. Litch, Patrick Musonda, Bellington Vwalika were omitted as an author in the author list. All members of The Global Alliance to Prevent Prematurity and Stillbirth (GAPPS) and The Alliance for Maternal and Newborn Health Improvement (AMANHI) are listed in the author list.

The original Article has been corrected.

