# Supplementary material for: Prediction of gestational age using urinary metabolites in term and preterm pregnancies
Source: Sci Rep. 2022 May 16;12:8033. doi: 10.1038/s41598-022-11866-6 (PMC9110694; doi:10.1038/s41598-022-11866-6)
Supplement: Supplementary file 1 — Supplementary Information 1. [file 41598_2022_11866_MOESM1_ESM.pdf]

Supplementary Figure 1

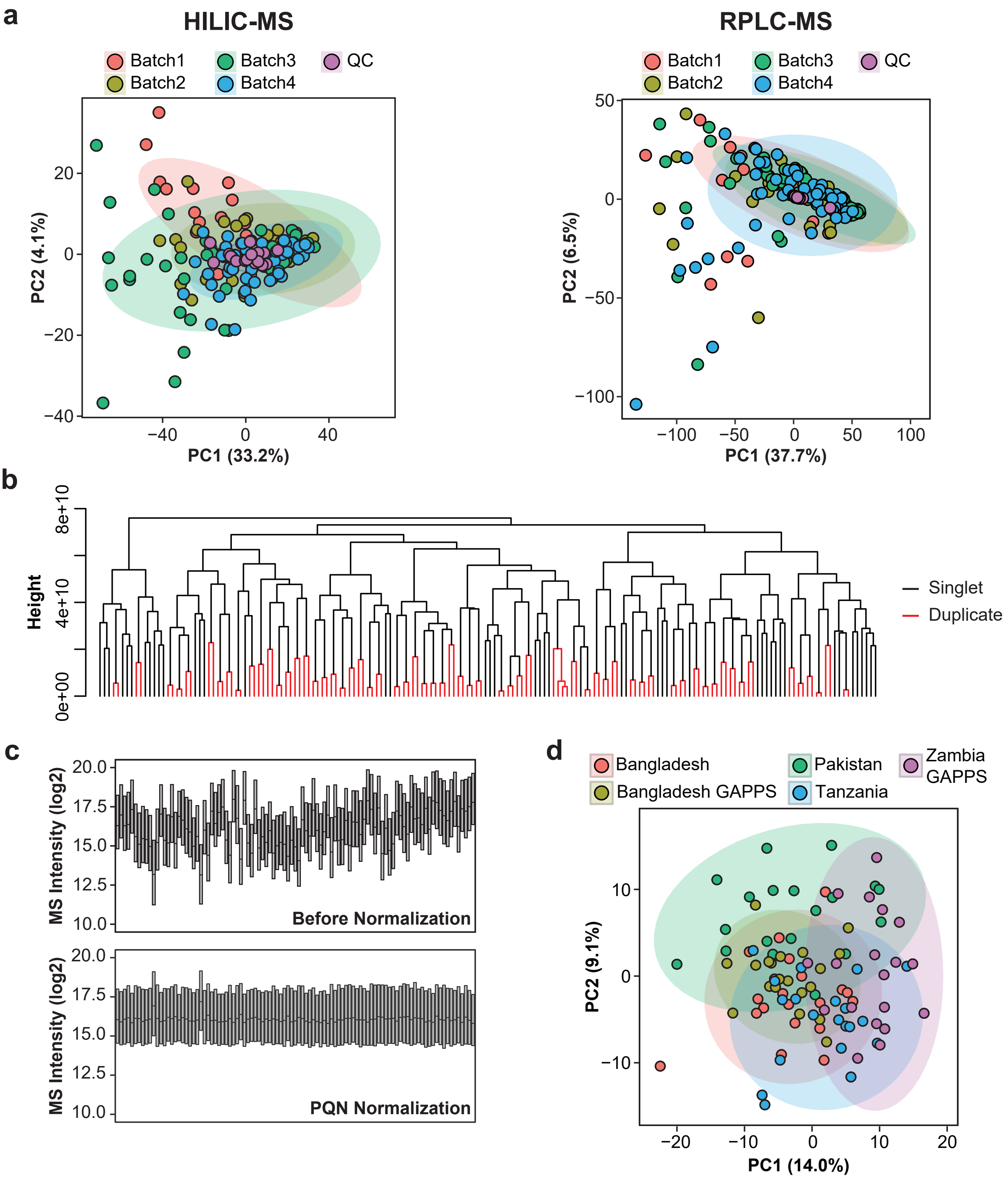

Figure S1. Quality controls for data generation, sample collection and handling. a) Principal component analysis plots of data generated in HILIC and RPLC modes. The study samples were intermixed suggesting limited batch effect and the QCs clustered together indicating good technical reproducibility. Each dot represents a sample colored by batch information. b) Hierarchical clustering of all the samples analyzed in the study (n = 172). Clustering distance = Spearman, clustering method = complete. Multiple aliquots for each sample were processed and analyzed in a random order. Branches in red indicate duplicate samples that present a tight clustering demonstrating the quality of the assay. c) Dilution effect correction using probabilistic quotient normalization (PQN). The distribution of MS signal intensity was variable across samples and became comparable after normalization. Of note, the two most diluted study samples became the most concentrated after normalization demonstrating an overcorrection of the MS signal and explaining why they were outliers in RF prediction models. d) Principal component analysis of all the samples in the study (n = 99) color-coded by collection sites. The study samples were mainly intermixed suggesting limited sample collection and handling variability across sites. Related to **Figure 1**.

Supplementary Figure 2

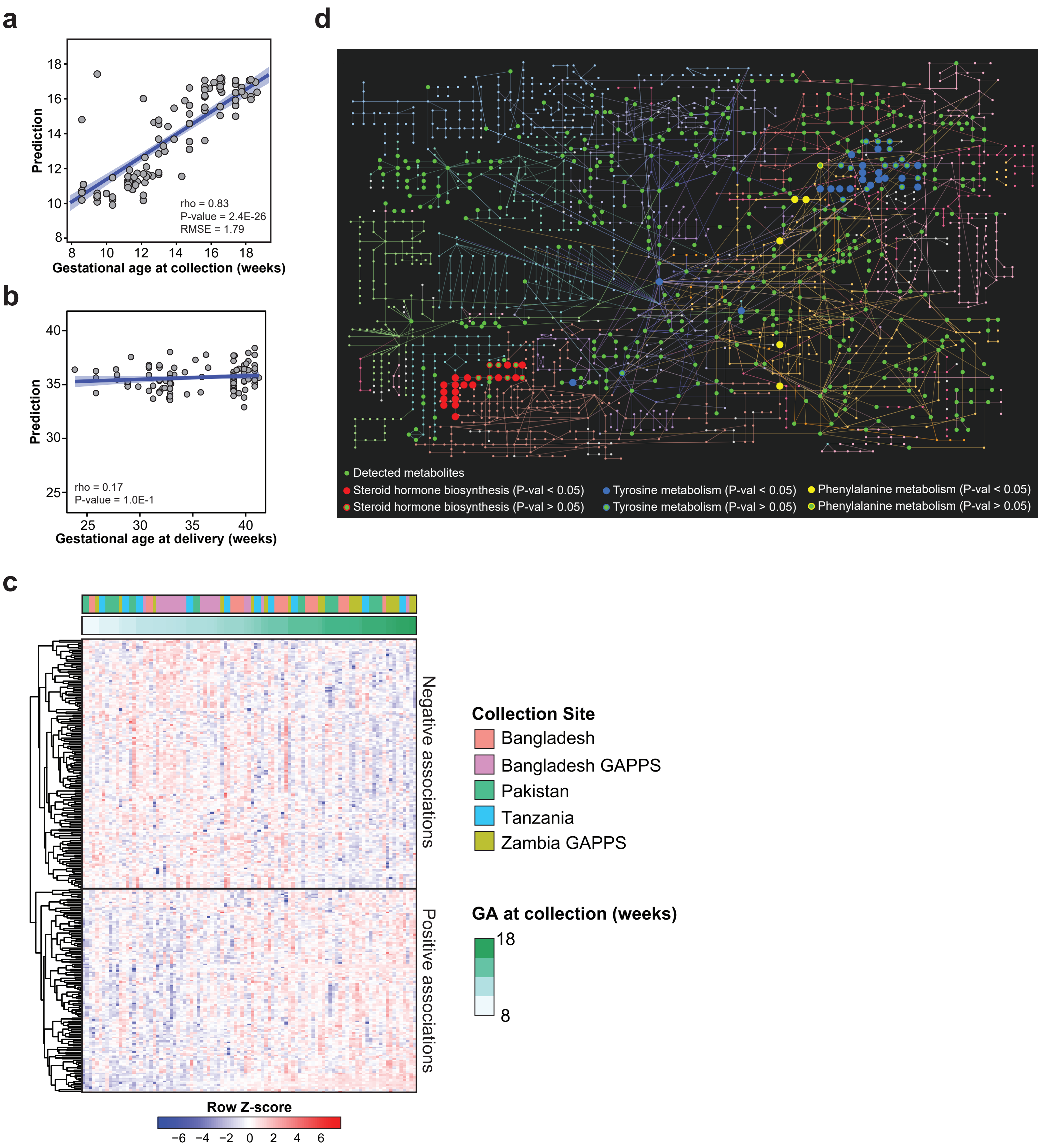

Figure S2. Prediction of gestational age at time of collection. RF modeling of GA at sampling (a) and at delivery (b) using all the samples in the study (n = 99). The blue area represents the 95% confidence interval. c) Heatmap of all the predictive metabolites showing positive (55%) and negative (45%) associations with GA. d) KEGG map showing metabolites involved in the pathways significantly enriched using metabolites selected in the RF prediction model of GA (P-value < 0.05)[35-37]. Related to **Figure 2**.

Supplementary Figure 3

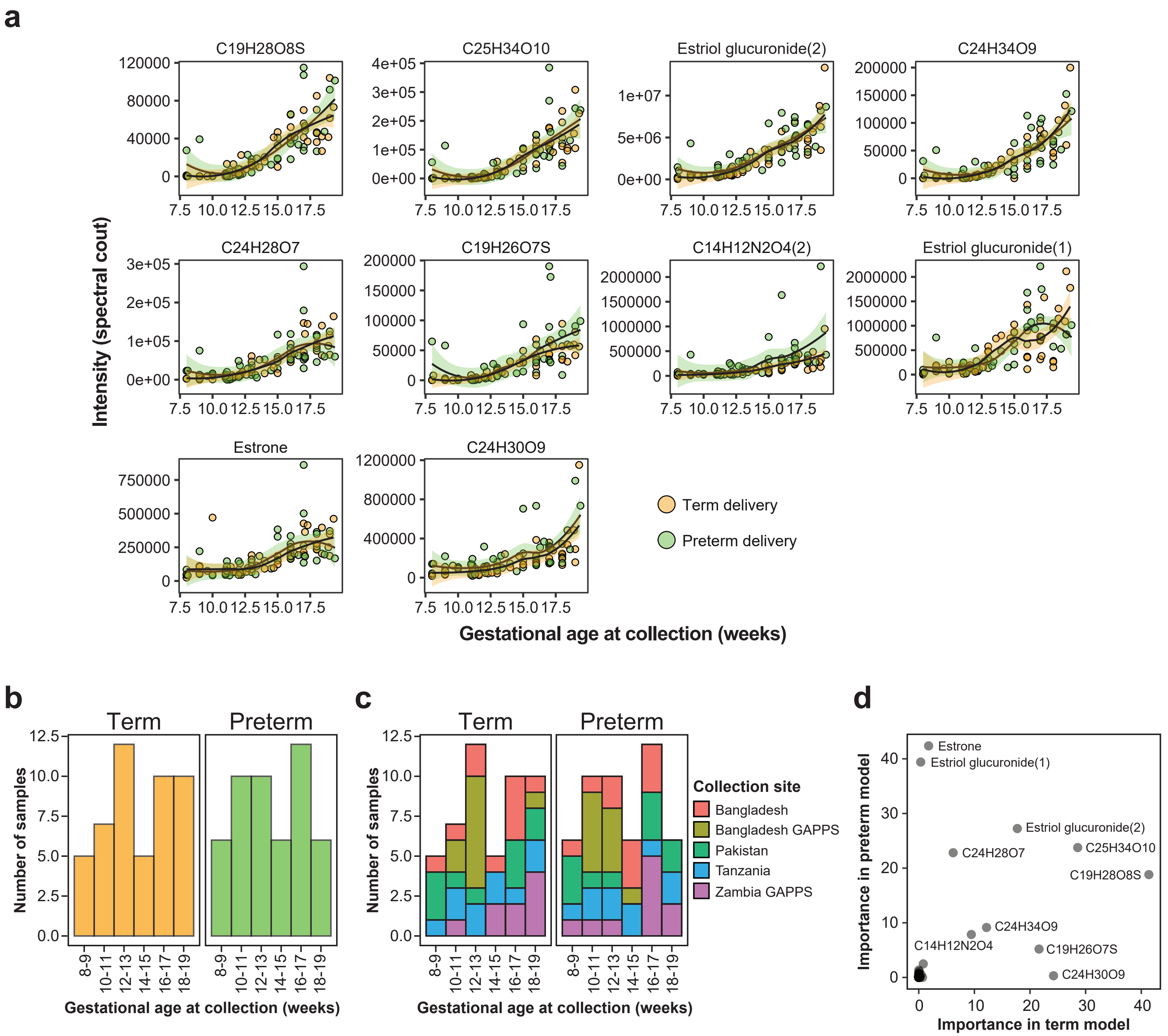

Figure S3. Prediction of gestational age at time of collection in term and preterm pregnancies. a) Top 10 metabolites in both term and preterm predictive models and LOESS fit. The shaded areas represent the 95% confidence interval. Number of samples (b) and origin (c) of samples across GA ranges. d) Importance of metabolites in term and preterm RF models. Related to **Figure 3**.
